# Bridging Algorithms and Biocatalysis: Perspectives on AI-Supported Enzyme Engineering

**DOI:** 10.3390/molecules31132359

**Published:** 2026-07-04

**Authors:** Rosa Teijeiro-Juiz, Thomas Brück, Bernhard Loll

**Affiliations:** 1 Laboratory of Structural Biochemistry, Institute of Chemistry and Biochemistry, Freie Universität Berlin, 14195 Berlin, Germany; rosa.teijeiro.juiz@fu-berlin.de; 2 Werner Siemens-Chair of Synthetic Biotechnology, TUM School of Natural Sciences, Technical University of Munich (TUM), 85748 Garching, Germany; brueck@tum.de

**Keywords:** enzyme design, protein engineering, artificial intelligence, protein stability, de novo design, non-canonical amino acids

## Abstract

The combination of computational and experimental methods has become indispensable for optimization and rational enzyme design. Recently, the development of artificial intelligence (AI)-based tools has further streamlined enzyme engineering pipelines, enabling more accurate designs, while reducing the number of variants required for experimental validation. However, due to the intricate complexity of enzymatic systems, significant challenges must be addressed before we take the next step to fully optimize the use of these AI-guided enzyme design methodologies. These challenges include un-curated datasets, the need to consider both the static and dynamic structure of enzymes, and the requirement for effective interdisciplinary collaborations to ensure the integration of computational and experimental approaches. Here, we present recent advances in AI-based computational enzyme design, discussing the main challenges in the field and how a combination with classical physics-based methods could help overcome them. We further explore novel trends that could completely modulate the future of protein design and provide our outlook on the key concepts and future opportunities that will shape the next steps of enzyme design.

## 1. Introduction

Enzymes serve as highly efficient catalysts mediating the acceleration of chemical reactions happening in all living organisms [1,2,3,4]. The precision and increased catalytic rate enzymes grant over the reaction outcome has motivated researchers to employ these proteins with the goal of overcoming some of the challenges emerging from traditional chemical processes [5]. In the last two decades, biocatalysis has become crucial for green and sustainable industrial biotechnological processes within biopharmaceutical, food or biofuel sectors, among others [6,7,8,9]. During these industrial processes, enzymes must resist extreme environments, ranging from high temperatures to non-natural pH conditions, or withstand organic solvents. These unnatural conditions could compromise the stability of the enzymes and hinder their ability to optimally catalyze and perform the reaction of interest [10]. Thereby, tailoring enzymatic systems for the catalysis of specific chemical reactions, sometimes under rather harsh industrial conditions, has become a main task for biochemists/biotechnologists.

Traditionally, researchers based novel enzyme design on available structural information of the respective protein. Enzyme function could rationally be altered by targeting specific amino acid residues, unlocking a desired change in the properties of the enzyme. Historically, this was performed through a directed evolution approach, which involves random mutagenesis on the gene of interest, to then screen and select enzyme variants that show improvement or the desired properties [11,12]. While this methodology has demonstrated positive results, directed evolution generates thousands of variants that then need to be experimentally validated, turning this process into a cost and time expensive approach for enzyme design [13,14]. More recently, the rise of computational approaches, which study dynamics and mechanics of the system, in combination with this traditional evolutionary method, has produced outstanding results, managing to design enzymes which, in some cases, could even rival the efficiency of natural catalytic proteins [15].

Current advances in AI, specifically in deep learning approaches for prediction of protein structure and sequence design, have transformed and accelerated our ability to accurately engineer biological systems [16,17]. By learning from curated databases, AI methods can predict which alterations in the sequence of the protein would allow for the improvement of different properties like stability or activity. However, despite the rapid progress in the protein design field, enzyme engineering remains challenging due to the complexity of enzymatic systems [18,19,20].

In this perspective article, we discuss the current advantages and limitations AI-guided enzyme design offers, highlight main challenges that remain unsolved, and outline emerging trends in the field that may shape the future of enzyme engineering.

## 2. Challenges in Predicting and Designing Enzymes

**Figure 1:**
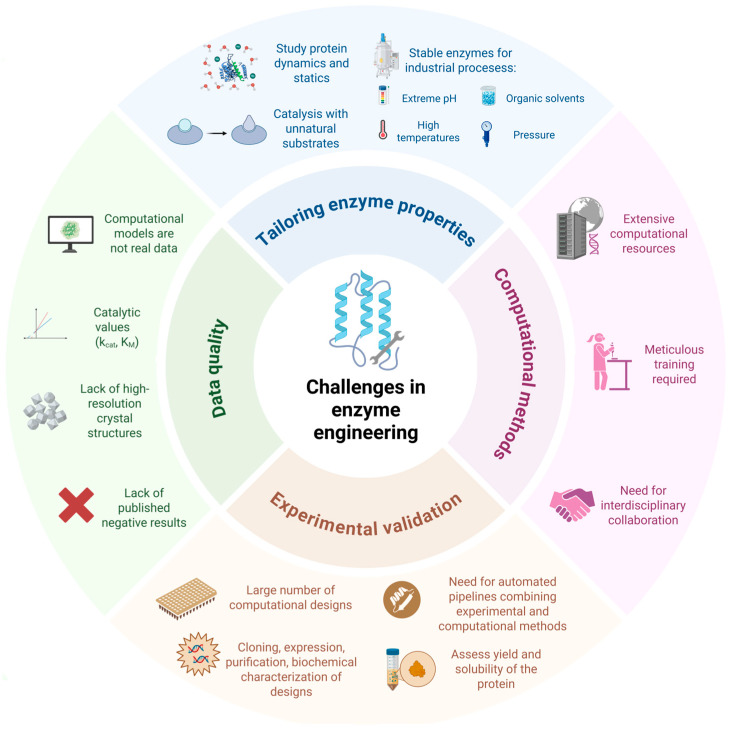
Current challenges in enzyme design for experiments, which can be sub-divided in for major categories: tailoring enzyme properties, computational methods, experimental validation, and data quality. The figure highlights that enzyme engineering is a multidisciplinary field where progress depends on balancing accurate data, advanced computational modeling, effective experimental validation, and the ability to tailor enzyme properties for specific applications. Overcoming these challenges is essential for developing robust enzymes for industrial, environmental, and biomedical applications. The data quality is important for training data sets for AI tools. Ideally, these data should include negative results. Based on the experimental data, transition state barriers could be calculated by MD or QM/MM methods. Protein or fold designs with increased stability and activity could be generated with AI-based tools such as AF3, ESMFold, or RoseTTAFold. The interaction with experimental biochemistry is important to validated to computer-based novel designs. This might include determination of the protein melting temperature to confirm an increased protein stability or for instance an enzymatic characterization of the kinetic parameters. Ideally, such experimental data flow back into the training sets.

Enzymatic systems are extensively complex. Holding a deep understanding of the structure and function of the biocatalyst is required, but not enough, for a complete rational and optimized enzyme design approach [14]. Earlier protein design approaches, as applied for the Kemp elimination, were based on computed transition state geometries [21]. The protein variants were generated by error-prone PCR. Recently, a fully computational workflow improved the catalytic activity of the Kemp eliminase [22]. The synergy of computational and experimental methods is imperative, and newly developed AI-driven tools are gaining traction due to their ability to drastically improve the accuracy when engineering new enzyme variants [16]. Despite all the advances and improvements in the design of biocatalysts, scientists are still facing a wide array of challenges [23,24,25]. In this section we want to focus on some of the major bottlenecks in the computational–experimental pipelines for enzyme design strategies (Figure 1).

One of the biggest challenges AI tools face is the lack of quality structural and functional data [26,27]. An elevated number of enzyme systems lack high-resolution crystal structures, and, while in recent years advances in structure prediction have provided scientists with the ability to predict enzyme models that can then be employed for enzyme design analysis, it is crucial to remember that in the end, these are just models, not real, verified, experimental data [27]. In the absence of proper comprehensive functional and structural datasets, how reliable are the trained machine learning (ML) models for enzyme design? Moreover, not only structural datasets are missing, but there is also a lack of standardized kinetic constants (*k*_cat_, *K*_M_) in the published datasets [28]. Accessibility to this information and the possibility of feeding these data into algorithms would potentially improve their predictive power towards enzymatic activity. In addition, there is a dataset bias towards successful variants, most frequently completely ignoring negative results and resulting in a data imbalance that can affect the algorithm’s predictions [29,30]. Feeding negative results into AI-driven tools would allow them to learn which changes in the enzyme do not bring the desired properties to the system. Without the incorporation of negative results, a model might have the tendency to become overly optimistic and may predict many proteins as active even though they are not. In general, inclusion of negative results could teach the AI decision making between success and failures. The reliability of AI predictions is ultimately constrained by the quality and diversity of available training data, which is why putting focus into obtaining more structural and functional data, as well as documenting negative results, should be a major focus in the future [31].

Since computational approaches have become a main component of enzyme design pipelines, multiple tools and algorithms have been developed with the goal of improving the rational tailoring of biocatalysts [16,32]. Some of these methods, apart from being time-consuming, also require extensive computational resources. Moreover, to use some of the more complex tools, meticulous training is required. For trained experimentalists who lack experience in computational software, it can be very tedious to properly learn how to use these tools. The increasing methodological complexity of computational tools creates barriers for experimental researchers, highlighting the need for public accessible platforms and interdisciplinary collaboration.

As previously stated, tailoring robust enzymes for biotechnological industrial processes is currently a point of interest within the field of protein design [33]. In order to withstand industrial conditions, such as extreme pH, high temperatures, organic solvents, or high pressure, enzymes must be stable enough, so that they do not lose activity or even completely denature [34,35]. However, one of the main challenges we currently face when designing industrial enzymes is the ability to, while increasing the stability of the system, maintain sufficient flexibility in the scaffold, for the reaction to commence. Thereby, another key focus point for the future of enzyme design is to combine engineering strategies that analyze both dynamics and statics of the entire protein, establishing relationships between stability and catalytic efficiency [36].

Following up the growing interest in enzyme design towards industrial biotechnology, another major bottleneck lies in the ability to control the substrate selectivity and specificity [37]. Due to economic reasons, or with the objective of obtaining novel products of the reaction, industrial biocatalytic processes occasionally rely on the usage of unnatural substrates [38]. However, predicting how enzymes will accommodate and transform different substrates remains challenging because of the complex relation between active-site architecture, protein dynamics, and reaction mechanisms [39]. Even minor structural differences in substrates can lead to changes in binding modes or catalytic efficiency [40]. Deep structural and chemical knowledge of the enzyme–substrate complex could allow for the prediction of the reaction outcome, yet current computational approaches still struggle to reliably capture these effects [41]. Addressing this limitation will be critical for expanding the applicability of enzyme design towards industrially relevant transformation involving unnatural substrates.

Finally, despite the rapid advances in computational prediction, experimental validation remains a challenge in enzyme design workflows. Computational prediction tools tend to generate large number of candidate-designs and structural models. Depending on the enzyme properties which are target for the optimization, translating this into experimental results might require intensive cloning, expression, purification and biochemical characterization [42,43,44]. Moreover, success rates are often limited by factors that are difficult to predict computationally, such as expression yield or solubility. Consequently, the development of high-throughput screening platforms and automated workflows that further combine computational design with experimental data, which ideally will be then fed back into the algorithms guiding the computational design, will be essential to fully exploit the potential of AI-driven enzyme engineering.

## 3. AI Tools Available for Enzyme Design

The revolution AI-guided tools have brought into the enzyme design field originates from their ability to optimize multiple properties simultaneously, including structure, stability and catalytic function. In contrast to traditional approaches, which often rely on experimental screening, AI methods enable the a priori exploration of sequence-structure–function relationships [45]. Rather than replacing established methodologies, such as directed evolution, these approaches expand the methodology for enzyme design and enable new opportunities for rational enzyme engineering. In this section we present a collection of AI tools which can help experimentalists guide their enzyme design strategies. For a more detailed comparison between these tools and when to use them, readers can refer to the cited reviews. More general reviews introducing the methodology of protein folding and design have been recently published [22,46,47,48,49,50,51].

### 3.1. Structure Prediction

As early as 1973, Anfinsen postulated that the amino acid sequence of a protein encodes for its three-dimensional structure [45]. The recognition of AI-based structure prediction by the 2024 Nobel Prize in Chemistry further highlights the impact of these approaches for protein design. The possibility of predicting the structure of proteins, and therefore, of enzymes, has completely changed the status quo of the field. In the past, researchers were limited by the vast number of designs which needed to be tested before finding one that would fold in the correct way [52]. Now, this can be reduced by utilizing AlphaFold3 (AF3) [53] predictions to estimate which designs have a higher probability of folding in the correct way. This model enables high-accuracy prediction of biomolecular complexes starting from polymer sequences, residue modifications and ligand SMILES. While AF3 presents some limitations, such as the output model not respecting chirality or the low accuracy of the prediction for certain targets, the results are still outstanding, managing to provide high-quality prediction that matches the later observed experimental structural data [54].

AF3 set an unprecedented revolution for protein structure prediction, but it is no longer the only available tool to study the folding of biomacromolecules. Lately, methods such as Chai-1 [55] or Boltz-2 [56] have emerged; they allow for the prediction of full biomolecular complexes through an open-source code. Both of these novel methods report computationally fast accurate results for structure prediction, and while they also present limitations, they must be considered as challengers for AF3. Another example of this newly developed structure prediction methods is RoseTTaFold All-Atom, created by David Baker’s group [57]. This AI-guided tool allows for the prediction of full assemblies and is also available open-source.

Overall, as previously mentioned, a major bottleneck in protein and therefore enzyme science is the lack of good, validated datasets [58]. Despite their role in advancing enzyme design, it is critical to remember that many of these functional prediction tools remain insufficiently validated experimentally, and their predictive power across diverse enzyme classes is still uncertain.

### 3.2. Function Prediction and Structure-to-Function Design

While structure prediction does not equal function prediction in protein systems, it remains true that both properties are heavily linked [59,60]. Therefore, another major interest in the enzyme design field is development of AI tools which can assist in the design of sequences that will enable the catalysis of a specific chemical reaction, allowing for the prediction of the enzyme function. Currently, a different array of AI tools is able to assist with the structure-to-function design. For the investigation of the substrate affinity of enzymes, researchers can make use of tools, such as FusionESP [61], EZSpecificity [62] or Catnip [63], the latest specifically being used for the α-ketoglutarate/Fe(II) non-heme iron enzymatic family. These computational methods do not present experimental validation, and therefore for the moment, the accuracy of the prediction is disputable.

Regarding the direct prediction of catalysis, tools such as SelenzymeRF [64], ALDELE [65] and CLEAN [66] can assist. While the latter focuses on the prediction of function for uncharacterized enzymes, ALDELE focuses on the prediction of the potential catalysis facilitated by an enzyme. SelenzymeRF, while also focusing on the prediction of catalysis, ranks candidate enzymes in order to provide information about the potential of each system to catalyze the reaction. Both SelenzymeRF and CLEAN have been experimentally validated, while ALDELE does not provide experimental data for the moment.

Finally, to accurately design a protein sequence which will eventually fold into an enzyme capable of catalyzing a specific reaction, efforts to develop AI tools to assist with this matter have also come up with different methods. EnzyACT [67] can potentially assist with the prediction of the impact single or multiple mutations happening in the active site may have on the enzyme. On a bigger level, Progen [68] is able to completely generate artificial protein sequences with a predictable function. Other tools like PLACER [69] can enable researchers to assess the accuracy and pre-organization of the active site. These last two AI-guided methods have been experimentally validated and are currently assisting in the accurate design of novel enzymes.

### 3.3. Optimization of Stability

Protein stability represents a critical parameter for enzyme performance, specifically for industrial applications that require robust enzymes which can catalyze reactions under non-physiological conditions [70]. Enzymes must balance two competing requirements: stability versus activity. The enzyme must maintain a correct fold and adopt a rigid structure under physiological or industrial conditions. On the other hand, activity includes the freedom to adopt different conformations and interactions that facilitate catalysis. In consequence, features that promote activity can destabilize the protein, while features that enhance stability may restrict the motions needed for catalysis. Notably, amino acid residues involved in catalysis are often placed in energetically unfavorable positions to stabilize the transition state. Mutations introduced to improve catalytic efficiency often can end up reducing the structural stability of the system [71,72]. Different AI-based stability predictors have arisen as valuable tools for the assessment of mutational effects on the stability of enzymes.

ProStab [73], ProstaNet [74] ThermoMPNN [75] and Pythia [76] are AI models that allow researchers to study the stabilizing and de-stabilizing effects of point-mutations in the target enzyme. The latter acts as a self-supervised learning model while the other two carry out supervised learning. While these tools have shown consistent experimental data related to their predictions, there also exists an array of other computational methods that, while not being experimentally validated yet, also show potential as predictors to study the thermostability of protein systems. Some of these tools include SPURS [77], ESMtherm [78], ProSTAGE [79], SPIRED-Stab [80] or Stability Oracle [81].

## 4. Integration of AI Tools and Traditional Physics-Based Methods

While AI methods are completely transforming enzyme engineering, bringing more accurate results, classical physics-based computational methods remain essential for understanding and predicting enzyme behavior at an atomic level. Techniques such as molecular docking, molecular dynamics (MD) simulations, and quantum mechanics/molecular mechanics (QM/MM) provide complementary insights to AI-driven models [82,83,84]. Rather than presenting these two approaches as competing methodologies, AI and physics-based computational design tools should be viewed as combinatorial components of modern enzyme design workflows.

Physics-based simulations offer the advantage of modeling molecular and atomic interactions using established physical principles, allowing the exploration of the system’s dynamics and energy [85,86]. Dynamics and energy data are critical for the assessment of enzyme catalysis as small energetic differences and transition states can determine catalytic efficiency and activity [87,88]. In the end, just predicting enzyme structure with AI-driven tools will only provide structural data, but knowing how this structure will behave during a reaction in a specific environment is equally important. Therefore, integrating mechanistic simulations with AI predictions can improve the accuracy of enzyme design pipelines.

### 4.1. Molecular Dynamics Simulations

MD simulations are widely used to analyze protein flexibility, substrate binding modes, stability and behavior under different conditions. Unlike static structural prediction, MD provides with time/conformation data, allowing researchers to capture motion that may be related with the catalytic activity of the system [89,90,91]. This is particularly interesting for enzymatic activity studies that depend on rare conformational states that are not evident from predicted structural models.

In enzyme engineering, MD simulations can assist for the evaluation of structural stability of designed variants, identifying flexible regions suitable for mutagenesis, and refining active site geometries predicted by AI-based tools [92,93]. However, MD simulations are computationally demanding and sensitive to force-field accuracy, which may restrict the application in large-scale screening workflows [94].

### 4.2. Molecular Docking

Molecular docking remains a commonly employed method for ligand binding mode prediction and for estimating substrate specificity in enzyme systems [95,96,97]. Docking approaches are computationally efficient and can rapidly scan large libraries of new designs, appearing as an interesting approach in early-stage design workflow. Nevertheless, traditional docking methods rely on simplified scoring functions and often treat proteins as rigid or semi-flexible entities, which can limit their accuracy when modeling enzymatic reactions that involve conformational rearrangements. On top of this, docking scores do not directly correspond to catalytic activity, as binding affinity alone does not capture transition state stabilization or reaction kinetics. These limitations highlight the need to interpret docking results cautiously and to complement them with higher-resolution simulations and, if possible, experimental validation [98,99,100,101]. Presenting the wide array of available docking methods is out the scope of this article, but readers can refer to the cited reviews for further information [95,97,102].

### 4.3. Quantum Mechanics/Molecular Mechanics Methods

QM/MM methods represent the most rigorous computational framework for studying enzymatic reaction mechanisms. By treating the reactive region using QM while modeling the surrounding environment with MM, researchers are able to investigate bond formation and cleavage, charge redistribution, and transition state stabilization within enzymatic systems [103,104]. QM/MM calculations have played a key role in investigating catalytic mechanisms and identifying key catalytic residues responsible for activity. In the context of enzyme design, these methods can guide the rational mutagenesis of catalytic residues, evaluate the effects of these alterations on the reaction, and validate AI-based predictions [105,106]. However, their high computational cost and methodological complexity limit their application to relatively small systems or focused studies rather than large-scale screening of enzymes.

## 5. Emerging Opportunities in Enzyme Engineering

So far, we have focused on some of the more traditional enzyme properties and processes and how AI tools can help tailor them, but of course as science advances, new opportunities come up in the field of enzyme engineering, opening doors for novel catalysis and design methods.

### 5.1. Unnatural Amino Acids

One interesting new addition to the field, is the possibility of utilizing non-canonical amino acids (ncAAs) for rational tailoring of enzymes. Traditionally, catalytic system design was limited to the use of 20 natural amino acids, but since introduction of synthetic biology methods that allow for the incorporation of unnatural amino acids into proteins, the possibilities are unlimited [107,108,109]. AI-guided design may be particularly powerful when combined with expanded genetic codes, enabling catalytic functionalities beyond those accessible to natural evolution. The wide library of available ncAAs offers the possibility to tailor enzymes very specifically. If, rationally, we believe the introduction of certain functional groups in the active site of the enzyme will allow for interesting changes in the reaction, but none of the 20 natural amino acids would be able to provide this functional group in that specific position, it is possible that one of the newly developed ncAAs can do this instead.

### 5.2. De Novo Design

De novo design of enzymes represents another rapid growing trend in the field of enzyme engineering. In contrast to traditional methodologies, de novo design creates new enzymes with pre-defined catalytic functions employing computational tools [19,110,111,112,113,114]. Generative AI approaches, including protein language models, hallucination methods and diffusion models, are reshaping the field. These methods allow us to generate backbone conformations optimized around functional motifs, catalytic residues, or transition-state geometries, facilitating the creation of enzymes to perform specific reactions [115,116,117]. Conditional design strategies, in which constraints or active-site geometries are predefined, coexist with unconditional approaches, which focus on generating entirely new folds without a starting scaffold.

Despite these advances, de novo design also faces challenges. While there have been cases of de novo designed enzymes that afterwards showed impressive experimental results [118,119], the reality is that generally the catalytic efficiency of these newly designed proteins has a tendency to be lower than in natural enzymes. Currently protein design works best for enzymes that have been very well characterized in terms of active site architectures, kinetics, transition state barriers. Often those enzymes like the Kemp eliminase have been already extensively studied by directed evolution or error-prone PCR [21,22]. This reflects the limitations of accurately modeling reactions from scratch. Moreover, generative models are trained on databases of naturally occurring enzymes, generating a biased exploration of sequence space towards naturally evolved proteins. In the future, it will be crucial to combine this approach with previously mentioned physics-based methods to bridge the gap between structural design and catalytic performance.

## 6. Conclusions and Future Perspectives

AI is rapidly transforming the field of enzyme engineering, providing unprecedented capabilities for protein structure prediction, sequence design, and functional optimization. However, despite these advances, achieving truly predictive enzyme design remains a significant scientific challenge. Enzymatic catalysis is determined by complex energetic functions, conformational dynamics, and environmental interactions that are not yet fully captured by current computational models. As a result, experimental validation and mechanistic understanding remains essential components of enzyme engineering workflows.

One of the key conclusions of the recent progress is that structure prediction alone is insufficient to enable reliable enzyme design. Future advances will likely depend on deeper integration of AI-driven tools with physics-based modeling approaches capable of describing catalytic mechanisms and protein dynamics. The combination of these computational frameworks with improved sampling strategies may significantly enhance predictive power of AI methods while preserving interpretability regarding the dynamics and activity of the system.

Another critical factor that will shape the future of AI-guided enzyme design is data availability. The development of standardized, high-quality datasets containing experimental structural data, kinetic parameters, stability measurements, and negative experimental results will be essential for improving model generalization and reliability. In parallel, advances in high-throughput screening are expected to enable full computational–experimental pipelines, accelerating the discovery of functional biocatalysts.

Emerging technologies are also expanding the conceptual boundaries of enzyme engineering. In the coming years, ncAA-based enzyme design is expected to play an increasingly central role in expanding the functional and structural repertoire of biocatalysts. Since experimental screening of ncAA-containing libraries remains both expensive and time-consuming, AI-guided modeling will become indispensable for prioritizing promising designs and building efficient, iterative engineering workflows.

As generative models and experimental screening technologies further advance, we can anticipate a transition from manual, case-by-case protein design toward more automated enzyme engineering systems.

Looking ahead, we hypothesize the next 3 years will see an exponential growth in the number of designed enzymes entering the market. The development of fully automated enzyme design pipelines will allow experimental researchers to employ complex computational tools and generate tailored de novo enzymes for specific applications. However, we also expect that a significant number of these designs will still not be fully functional, as fully reproducing the complexity of enzymatic catalysis is still beyond the capabilities of current computational methods. Therefore, we believe that, while enabling researchers to more accurately tailor enzyme engineering, AI tools will never be able to replace human expertise. Specialists in molecular dynamics, catalysis and experimental validation will remain essential to interpret the designs, optimize them and assess their functionality.

Ultimately, the future of enzyme engineering lies not in AI alone but in the synergistic combination of data-driven algorithms, physical principles, and experimental innovation. As these components continue to evolve together, the prospect of designing enzymes with tailored activities, novel chemistries, and industrial robustness may become an achievable reality.

## Data Availability

No new data were created or analyzed in this study. Data sharing is not applicable to this article.

## Abbreviations

The following abbreviations are used in this manuscript:

AI	artificial intelligence
AF	AlphaFold3
MD	molecular dynamics
QM/MM	quantum mechanics/molecular mechanics
ncAAs	non-canonical amino acids
